# Life stories of young women who experience rejection from their mothers

**DOI:** 10.4102/curationis.v38i1.1429

**Published:** 2015-08-12

**Authors:** Selina C. Mosman, Marie Poggenpoel, Chris Myburgh

**Affiliations:** 1 Department of Nursing Science, University of Johannesburg, South Africa; 2 Department of Educational Psychology, University of Johannesburg, South Africa

## Abstract

**Background:**

When a daughter perceives rejection from her mother, she is bound to be sensitive to rejection for most if not all of her life. Such an experience influences almost all future relationships.

**Objectives:**

The purpose of this research was to explore and describe the life stories of young women who perceived rejection from their mothers and to formulate guidelines to assist them.

**Method:**

A phenomenological interpretive method that is explorative, descriptive, and contextual was used to explore everyday life experiences. Network sampling was used. In-depth phenomenological interviews were conducted with the young women so that they could define the most important dimensions of their life stories and elaborate on what is relevant to them. They were asked: ‘Tell me your life story.’ One of the authors also had a life story of perceived maternal rejection; hence an auto-ethnography was critical and was included in the study. Thematic data analysis was applied.

**Results:**

Themes that emerged from the data were that the young women: (1) perceive ongoing challenges in forming and sustaining relationships in their lives; (2) experience their lives as conflicted because their relationship with the central core of their existence, their mother, is perceived as tumultuous; and (3) experience fundamental links to be missing in their ‘mother-daughter relationship’.

**Conclusion:**

Only a few women were interviewed regarding perceived rejection from their mothers. Further research in this regard is imperative.

## Introduction

A daughter's bond with her mother is a critical, quintessential one because their confidence is fragile and they understand themselves better through their key relationships, especially as they pass through key developmental stages such as adolescence and young adulthood. They become more self-aware and need plenty of attention at these stages as they are faced with questions of self-identity (Hartley-Brewer 2001:124). However, if a daughter perceives that her mother rejects her in her time of need, she loses her self-confidence, questions her identity and becomes further confused and unsettled by the altered behaviour of those she relies on.

Numerous studies have shown that a perceived poor relationship with a parent together with memories of perceived rejection may both directly be linked to the development of depressive symptoms right through to adulthood (Rholes & Simpson 2004:418). Jay (n.d.) mentions that when a daughter perceives that she is forced to go through life without the love, support and nurturing of a mother, she will grope blindly in the dark, crashing into walls and at times faltering and falling, unable to stand up again. Given this notion, we are of the opinion that there could be nothing more harmful to a daughter's self-belief, spirit of life and soul than perceived rejection from her mother.

### Problem statement

A fact sheet (Mind for better mental health 2009:n.p.) conducted in the United Kingdom on women and mental health indicates women's proneness to mental health challenges when they do not have positive coping or survival strategies to help them deal with these challenges. The fact sheet records rates of anxiety and depression as one and a half or two times higher in women than in men, with rates of self-harm being two or three times higher. The fact sheet states that at least one new mother in 10 would experience postnatal depression. It also mentions that out of the 1.15 million people who suffer from an eating disorder, 90% were found to be women (Mind for better mental health 2009:n.p.). It further suggests that these facts are often the outcome of ‘internalising’ negative emotions, such as the perception of parental rejection.

Rohner and Khaleque (n.d.) point out that considerable research literature shows that the quality of a parent-child relationship characterised by perceived parental acceptance (perceived love) or perceived parental rejection (perceived lack of love) is a major predictor of psychological functioning and development for both children and adults universally. Gull, Tanq and Malik (n.d.) report that persons who have perceived parental rejection are more likely to be hostile, dependent, emotionally unstable, emotionally unresponsive, to have impaired feelings of self-esteem and self-sufficiency, and to have a negative view of the world.

Lessesne and Kennedy (2005:757) suggest that poor mental health could contribute to cycles of intergenerational transmission of risks, leading to poor mental and physical health in children of exposed women. It states that if left untreated, mental, emotional and behavioural health concerns can greatly affect the development of a child, just as they affect the lives of adults. This means that if the daughter's perception of rejection from her mother is left unresolved, it can also affect the next generation. To be more precise, the daughter can predispose her child to the same mental, emotional and physical challenges that emerge from perceived parental rejection, if not worse. This can continue for generations and generations until the cycle is broken by effectively managing the phenomenon.

Because a person's quality of life depends greatly on his or her mental health, the ongoing existence of aspects that affect the mental health could greatly diminish that quality of life. The World Health Organization (2004:21) also relates the intrinsic value of mental health, describing it as a pivotal resource for persons, families, communities and nations and that it contributes to the social, human and economic capital of every society. This boils down to my (one of the above authors) concern that denying the young women mental health would be like denying them the need for survival, because mental health encompasses productive functioning and the capacity to build and maintain a fulfilling life.

The greatest issue here is that if anyone ought to love a child, it is a mother, as mothers are seen as life givers and the most fundamental teachers of survival skills (Mark n.d.; Stuart 2009). Any society's faith rests on mothers to bring up children who are productive and contribute positively to its development. Stuart (2009) even attests to some women who have perceived rejection from their mothers, once grown and being adults with families of their own, still referring to a mother's perceived rejection in their early years as the most disastrous experience of their lives.

#### Background

The relationship between a mother and a daughter is vital from birth to adulthood, as they share a bond that is unlike any other because of the symbiotic connection and natural liaison associated with this union (Stuart 2009). Interpersonal closeness with a mother is an integral part of mental health and a primary source of happiness, says Deeba (2001:1). If a girl grows up with the perception that she is being rejected by her mother through, for example, heavy criticism, she is likely to seek acceptance elsewhere. She often turns towards drugs, sex and other addictions to comfort herself (Jay n.d.).

A number of longitudinal studies show that perceived parental rejection often precedes the development of depressive symptoms. Many cross-cultural findings in support of this suggestion are available from many countries from around the world, including China, Egypt, England, Finland, India, Japan, Norway, Pakistan and elsewhere (Rohner & Khaleque n.d.). This suggests that a vast number of research studies exist on the effects of perceived rejection. However, more research studies need to be conducted into the results of those effects.

#### Research objectives

The overall research purpose was to explore and describe the life stories of young women who have perceived rejection from their mothers, and to formulate guidelines and recommendations that can assist them in coping with the challenge of their life stories.

### Definition of key concepts

**Life stories:** McAdams (2009:11) explains that life stories are narratives of the self that are internalised and evolving. Life stories will reveal how the young women who have perceived rejection from their mothers integrated their past, present, and future in a manner that displays some sense of their life meaning and purpose.

**Young women**: Sadock and Sadock (2007:45) mention that young adulthood is considered to begin at the end of adolescence and end at the age of 40. *The Oxford Advanced Learner’ s Dictionary (*2011:n.p.) defines women as being of the sex that can bear offspring or produce eggs. In relation to these clarifications, our research study involves young women aged between 18 and 35 years. The terms are used interchangeably with ‘daughters’ in our research study.

**Perceived:** Uys and Middleton (2004:836) define perceptual processes as the experience of sensing, interpreting and comprehending the social world through conscious awareness of the elements in it. Our research study evolved around the perception of rejection as it influences the way in which the young women under study thought and behaved.

**Rejection:** Rejection was applied in our research study as defined by Williams, Forgas and Von Hippel (2005:47) – the broadest, most generic term for instances where the young women perceive their relational value with their mother as being lower than they desire. Occurrences of either prior belonging or non-belonging are accompanied by the young women's either positive or negative evaluations of the mother, with or without the involvement of actual disassociation, and as a result of either comparative or non-comparative judgements. The term was used cohesively with perceived neglect in our research study.

#### Significance of the study

The significance of our research study lies in hearing and understanding the life stories of young women who have perceived rejection from their mothers. The perceived rejection by their mothers influences their lives, as they experience ongoing challenges in forming and sustaining relationships. The young women also experience that their lives are conflicted as their relationship with their mother is perceived as tumultuous. They experienced that even as they went through something painful in their lives, their mothers were not there for them. Because of these experiences and perceptions, these young women have low self-concepts that make them vulnerable to mental health challenges such as academic failure, sexual misconduct, depression and suicidal ideas. To address these mental health challenges these young women need to be facilitated to practice mindfulness in their everyday lives.

## Research design

### Design

A post-modern constructivist approach (Gray *et al.* 2007:184) was applied, where our interests were solely based on the young women's life stories of perceived rejection from their mothers as well as their settings, rather than on concepts and theories. The approach followed a qualitative, exploratory, descriptive, contextual research study design (Creswell 2013:59). This was because qualitative research requires us to get to the heart and soul of the young women's life stories and settings without imposing any expectations (Chambliss & Schutt 2010:10). Exploratory research is concerned with discovering and gathering new information about and insights into the phenomenon under study (Gray *et al.* 2007:37). Descriptive research has as its main objective the accurate and detailed portrayal of the participants’ beliefs, values, attitudes and cognitive processes that underlie the phenomenon under study (Polit & Beck 2010:552). Contextual research is when the natural setting in which events occur is used as an ‘observation post’ from which data are gathered (Gorman & Clayton 2005:4).

#### Population and sampling

Young women from surrounding townships in Johannesburg whose ages ranged between 18 and 35 years formed the population in our research study. The network sampling method (Burns & Grove 2005:353) was established in our social and professional circles. The criteria which were used for inclusion were as follows: they must be young women aged between 18 and 35 years; they must be from any township within Johannesburg; they must have perceived rejection from their biological mothers; they must be able to speak and fully understand any of the 11 South African languages; and they must give informed written consent to participate in our research study. An exclusion criterion was that the young women should not be unable to tell their life stories on perceived rejection from their mothers.

Data saturation was achieved after eight participants were interviewed, when no new information and repeating themes were evident in the interview data.

#### Data collection

A phenomenological interpretive method was used to relay the life stories of young women who have perceived rejection from their mothers. This method allowed us to set aside our own experiences and take on a fresh perspective on the phenomenon under study. According to Alaszewski (2006:36) accessing the participants’ interpretations of their world was the only way to achieve this. In-depth phenomenological interviews were conducted and audio taped to help recapture the responses and how they manifested. Informed written consent was obtained beforehand. One central question was posed to all participants which was ‘Tell me your life story.’ Observations were made and field notes were compiled because they provided us with a record of the experiences, activities, interactions and reflections of what occurred during the data collection process (Gorman & Clayton 2005:185). Data were collected until saturation point was reached. Transcriptions were done verbatim. An auto-ethnography was included to ensure that the data collected were unbiased and uninfluenced by the researcher who had a life story of perceived maternal rejection (Ty & Verduyn 2008:4).

#### Data analysis

Data were analysed by means of thematic coding (Creswell 2013:61–62). We went through the transcriptions of the interviews and highlighted units of meaning pertaining to the participants’ experience of their perceived rejection by their mothers. These units of meaning were then combined in significant statements to form themes. The themes were then used to write a description of what the participants experienced, with supporting direct quotations from the participants. Lastly the results were discussed in relation to the existing literature.

## Ethical considerations

It is our moral duty to conduct a research study that is ethically sound because ethics are concerned with morality as well as the balancing and respect of relations, rights, values and needs between the participants and researchers (Dhai & McQuoid-Mason 2011:147). Great care was applied in order to secure the well-being of the participants as the phenomenon under study may be a sensitive issue to them (Long & Johnson 2007:11). Before obtaining informed consent from the participants, their legal capacity to give consent and to comprehend all the elements involved in the subject matter being studied was first ensured (Burns & Grove 2005:193). We provided an explanation of our research study and its context prior to providing the consent form in order to equip the participants with efficient and enlightened appreciation of what they were consenting to (Long & Johnson 2007:9). The participants were informed that the information they shared would not be divulged to any other person without professional capacity for involvement in the research study (Long & Johnson 2007:16). The participants were also informed that the audio tapes and diaries used will be kept under lock and key and destroyed two years after publication of the research study. The participants were informed of their right to withdraw from the study at any time that they wished, notwithstanding our appreciation of their willingness to share their life stories.

All participants signed a consent form that afforded us permission to interview them and to audio tape their life stories. We undertook to attain the necessary skills needed to carry out effective interviews by means of the Masters in Psychiatric Nursing Science Programme, since interviews can be invasive to the psyche and are capable of posing risks to the health of the participants (Burns & Grove 2005:541). Our research study was approved by the Faculty of Health Science Academic Ethics Committee, University of Johannesburg (ethics clearance number AEC 22/01-2011). Participants were offered the telephone number of the Primary Health Clinic that serves the area they reside in if they needed any further support after verbalising their life stories about their perceived rejection from their mothers.

## Trustworthiness

The criteria of credibility, transferability, dependability and confirmability of our research study were addressed in an effort to present a convincing case that our work is academically sound (Shenton 2004:22). The following series of actions were carried out to ensure credibility of our research study: prolonged engagement, persistent observations, triangulation, reflexive journal, member checking and peer group review. Transferability was ascertained through a dense description of the demographics of the participants and a dense description of our research study's results, which included direct quotations from the participants (Polit & Beck 2010:503). The following measures were performed to achieve dependability of our research study: stepwise replication of the research method, dense description of the research method, code-recoding of data, and a dependability audit. The more formal approach of a dependability audit of the whole research process was carried out through a chain of evidence, and involved scrutiny of data by an external reviewer (Polit & Beck 2010:502) in order to establish confirmability of our research study.

## Results and discussion

Eight young females participated in this research. Their ages ranged from 18 to 35 years of age. Three of the participants were unemployed, two had permanent jobs, two were students, and one was a businesswoman. Three participants did not have matric, one was at university, two had matric and two were college students.

The findings of our research study were discussed under headings of the central theme, themes and categories that were derived from the process of data analysis. Three themes emerged from the life stories of the participants, under which three categories were identified from each. Verbatim extracts were included to illustrate each of them.

In terms of the central theme of the study, the young women perceive ongoing challenges in forming and sustaining relationships in their lives, which related to their low self-concept. The young women also experience living conflicted lives and perceive that there are fundamental links missing in their relationships with their mothers.

After prolonged engagements with the participants, similar challenges which they seemed to experience from their life stories of perceived rejection from their mothers seemed to stem from one common denominator, which we identified as low self-concept. To identify this, here is some of what the participants said during the interviews: ‘I’ve always been the black sheep’ (Participant 3), and ‘I feel as though I’m a failure because I failed to make her happy’ (Participant 1).

Self-concept holds a powerful function in mediating human behaviour, as it is thought to be a necessary element of health, happiness and the productivity of all individuals (Marsh, Craven & McInerney 2003:96). Self-concept is also vastly shaped by memory and personal experience because it is guided by what we pay attention to as well as the meaning we make of it (Brewer & Hewstone 2004:10). Many societal ills can be traced back to low self-concept, often resulting in personal and social ineffectiveness, as reported by Marsh *et al.* (2003:98). Amongst these challenges are academic failure, sexual misconduct, depression and suicide.

### Theme 1: Ongoing challenges in forming and sustaining relationships in their lives

The first and most fundamental pattern of relationship that a human being can experience, often associated with the one developed with a parent or a caregiver, often sets the tone for future relationship patterns (Uys & Middleton 2004:31). That being said, we found that the young women with life stories of perceived rejection from their mothers also perceived challenges in forming and sustaining other relationships in their lives.

The participants’ perceived hardships with parenting were found to be emotionally based, as we gathered that they were experiencing some or other burden in caring for their children. These are some of their statements: ‘I didn’t have a connection. It was difficult for my mind to adapt that now you have a child. I knew I was pregnant but it was hard …’ (Participant 8), and ‘I didn’t really like my son that much. Even after giving birth, I didn’t carry him …’ (Participant 2).

The emotional state of a parent is said to have a significant effect on the quality of the relationship that they build with their child (Bornstein *et al.* 2003:133). Shaffer (2005:144) explains that the young women most probably start out with the best intentions when they themselves become parents. They vow never to do to their children what they perceive to have been done to them – ‘but they often expect their infants to be “perfect” and to love them right away’ (Shaffer 2005:144). So when their babies are irritable and fussy, as all infants sometimes are, the already emotionally insecure young women are likely to feel rejected once again and may withdraw their affection, to the point of repeating the cycle of perceived parental rejection or neglect with their own child. Cottle (2003:24) gives another explanation, that the young women grow up to be parents that practice the same parenting patterns inflicted on them because they may know no other forms of interaction or attachment.

The participants spoke of abuse or being taken advantage of in relationships which they were in at the time or had been in. Although speaking of pain and unhappiness that they experienced in their intimate relationships, we identified that they somehow found themselves still attached to their partners. These are the words of some of the participants:

‘… I called him last week telling him about the baby and he was like, “Why didn’t you kill that baby because today I’ve got my own budget and I’ve got my own family to take care of”…’ (Participant 4)

‘… And this one day he said I should accompany him to his place and I did, and he actually raped me, twice. And he said he's sorry …’ (Participant 3)

Parental response that is perceived as sensitive is associated with secure attachment patterns, but because the young women perceive that their mothers failed to be sensitive and responsive to their needs, they start to develop insecure attachment and low self-worth (Miller 2004:69). With insecure attachment the young women may come to believe that they are bad and unworthy of love, respect and support (Sedikides & Spencer 2007:26). The young women may also develop a tendency of wanting to repair their low self-worth by seeking reassurance and validation from others (Miller 2004:288).

The participants in our research study were of the opinion that they related better with male friends than female friends. They stated their reasons based on their history and experiences with female friends, for example: ‘… I make better friends with guys …’ (Participant 7), and ‘… Okay, so there's these guys that I chill with, I don’t have that many girlfriends …’ (Participant 1).

Shaffer (2005:380) states that individuals who perceive neglect or rejection from their parent(s) often display a number of serious problems, including social anxiety. This is, at most, a result of the negative feelings and emotions that accompany their low self-concept. The shame and embarrassment that, conversely, accompanies low self-concept may inform the young women that they are at risk of group rejection (Sedikides & Spencer 2007:197). This may ultimately predispose them to self-compromising behaviours of either a desire to appease others for the sake of friendship or avoiding any closeness because of a negative view of people in general, especially other women (Miller 2004:70).

### Theme 2: Conflicted lives because their relationship with the central core of their existence, their mother, is perceived to be tumultuous

The young women experienced that their lives were conflicted because their life stories of perceived rejection from their mothers had not been dealt with and resolved (Uys & Middleton 2004:270). This seemed to have resulted in the young women's perceptions and emotions centred on their life stories festering in their lives and the organisation of their relationship with their mother. The categories identified under this follow below.

During the in-depth interviews with the young women the interviewing researcher experienced that they harboured a lot of anger towards their mothers, and as a result a sense of loss of respect for the mother could also be identified. Participant 3 said ‘It makes me have an evil heart. I have a lot of anger …’, whilst Participant 5 stated as follows:

‘I’m serious, I feel that way about my mother. I can’t say I know it's wrong … It's me being honest with my feelings … I can’t stand her, I can’t be with her, I can’t breathe the same air with her, I can’t live in the same space with her – I just can’t, I cannot! If it were up to me, I would be on one continent and she would be on the other right now. That's where I’m at with the way I feel about my mom.’

The rejection and harsh treatment perceived by the young women from their mothers are the core elements and socialising practices that contribute to the development of the anger that they harbour (Miller 2004:68). The anger can turn to hostility and resentment, as it is directed towards someone who is perceived to have threatened their identity and sense of security (Sedikides & Spencer 2007:190).

Even without asking about it, the young women spoke of their love for their mothers. Each time it was a vulnerable sight to see. The interviewing researcher also heard them speak of putting their mother's feelings first before their own, yet they feel a strong need to live away from her. Living away from her, however, proved hard for the young women, who still apparently had love for their mothers. These were some of their statements: ‘… I still can’t leave her, and I won’t. For some reason, she's got this hold on me [*crying* ]. But it's okay, it's hard …’ (Participant 6), and ‘… I love my mom very much. I don’t think she realises that I love her …’ (Participant 2).

The young women's apparent love for their mothers, despite perceiving rejection from them, mainly stems from the young women's need to protect the mother from the same hurt they feel from the whole perception of rejection (Miller 2004:74). The young women therefore tend not to want to inflict or cause their mothers the same pain, ultimately putting themselves in positions where they make sacrifices for her. However, they have a need to live away from the mother because being around her seems to be a reminder of the unpleasant feelings and emotions that the young women carry with them and, most importantly, what they believe about themselves (self-concept) and their relationship with their mothers (Coleman & Kerbo 2006:49).

One of our findings was that the young women seemed to be battling to get over the pain of their life stories of perceived rejection from their mothers. They cited the perceived negative behaviours that they saw themselves doing in trying to cope with or even forget about their situation. What was conflicting about this is that the young women believed that their life stories made them strong individuals, in that they could deal with any situation without necessarily relying on their mothers – yet they seemed to struggle with coping and survival strategies, even when it came to other pain in their lives. Here is some of what the participants said during the interviews: ‘Smoking, that's the one thing I can say I do when I’m stressed. I do smoke’ (Participant 4), and ‘I still have suicidal ideations because I can’t figure out if my mom really loves me …’ (Participant 8).

The young women tend to lose self-control and engage in self-defeating behaviours as a result of their life stories of perceived rejection from their mothers, as they lack the ability to regulate their emotions (Sedikides & Spencer 2007:320). Shaffer (2005:320) adds that many of those individuals who have perceived rejection or neglect are, however, remarkably resilient because of having had to be self-reliant in finding a perceived positive relationship elsewhere for themselves. However, this still does not remove the low concept of self that results from the perceived negative relationship with the mother, since she (the mother) remains her parent, an individual of high importance and influence in her life (Bornstein *et al.* 2003:244). Hence the challenge that the young women still encounter with coping and survival strategies when they face or think about their life stories of perceived rejection from their mothers.

### Theme 3: Fundamental links are perceived to be missing in their ‘mother-daughter relationship’

Young adulthood mostly requires establishing an identity that matches one's young adult goals (Sadock & Sadock 2007:45). This process can be viewed as an elaboration of self and is often influenced by relationships that the young adult regarded as important. If these relationships are perceived to be distorted, such as in the young women's life stories of perceived rejection from their mothers, the young women's inner definitions of themselves can become emotionally affected by how they view themselves and their relationship with their mothers. In line with this, the categories identified from the in-depth interviews with the young women are outlined below.

The participants experienced that even as they went through something painful in their lives, their mothers could not be there for them. What we also noted was that such memories seemed to be etched into their minds, and still appeared to be vivid and painful as they shared the experience. Participant 3 stated ‘I stay quiet and sometimes find myself crying at night when everyone is asleep knowing that no-one else can see or hear me’, whilst Participant 5 said:

‘I keep having these flashbacks of a cousin of mine touching me inappropriately. So I told my mother about it and she flipped out because to her I’m just mean and I lie and I just say things, because she doesn’t really know me.’

It is a parent's privilege to support their child and to satisfy a wide range of his or her emotional needs, since parental warmth and support are seen as key components in the development of a secure parent-child relationship (Coleman & Kerbo 2006:53). However, when a mother's reaction to her daughter's emotions is perceived to be negative, it denies the daughter an environment where she feels free to experience and express emotions (Miller 2004:404).

The young women in our research study perceived that their mothers failed to guide them through life and to establish a relationship where they could feel free to openly communicate with them about anything and everything. Some of them had the following to say during the interviews:

‘I remember my first menstrual cycle. I actually didn’t know how to use a sanitary towel. The sticky side that is supposed to be on the underwear, I would actually put it upside down …’ (Participant 7)

‘I’m afraid to tell her about a lot of things that are happening with me, she's harsh. I’m not able to sit down with her and talk about anything or my experiences at this age, being a single parent and also depending on her sometimes.’ (Participant 3)

Positive guidance is necessary in rearing a child because it instils structure and order that make the child's life easier in gaining a better understanding of the world and developing some sense of self-guidance (Miller 2004:70). Another factor that contributes to the development of self is how and how much a parent communicates with their child (Sedikides & Spencer 2007:77). Communication allows the parent to explain consequences of behaviour and reasons for rules which, ultimately, can become useful for the young women in reducing and/or eliminating maladaptive behaviours.

The young women were of the perception that, ever since they could remember, their mothers showed an inability to emotionally connect with them as there were no hugs, kisses or any signs that could assure them of her love or affection towards them. Some of the young women had the following to say in light of this:

‘I remember once as a child, because I grew up [*in* ] a very westernised way where kids hug and what, and I once tried to do that and she pushed me away. She just didn’t want that.’ (Participant 6)

‘I generally do not cry. It's a sign of weakness. That's what my brother told me and I never cried. And if my mother was there for me, she would have taught me to cry when I need to cry. That's how I feel. She wasn’t there for me, emotionally especially.’ (Participant 5)

A mother's perceived inability to provide love, affection and nurturing indicates a perceived failure to be responsive to the needs of her daughter. Such perceived insensitive parental responsiveness is likely to rob the daughter of the basic need for security and parental-affirmation, resulting in the daughter developing a negative self-identity (Miller 2004:68). A negative self-identity predisposes the daughter to insecure attachments and negative connections with both adults and peers (Shaffer 2005:437).

### Practical implications

A phenomenological approach was used to detail the many emotions and issues that the young women who have perceived rejection from their mothers endured. Our research study also displayed cause and effect from the phenomenon under study, which depicted a generational cycle of the phenomenon if left unmanaged. Guidelines and recommendations were formulated as a framework to assist the young women in coping with the challenges of their life stories.

## Limitations of the study

Our research study, although sensitive, was interesting and unique as not many studies had been done on the phenomenon under study, especially in South Africa. However this generated major challenges for us since there were not many theories to compare and contrast the findings with.

## Guidelines and recommendations

After having explored the life stories of young women who have perceived rejection from their mothers in order to understand the contributing factors thereto and consequences thereof, guidelines and recommendations were formulated to assist the young women with coping. In facilitating management of the ongoing challenges perceived by the young women in forming and sustaining relationships in their lives, the following guidelines were suggested: emphasis on the importance of parental socialisation; introduction to programmes that facilitate the importance of self-affirmation; and the need for a comprehensive approach to develop positive self-esteem. In facilitating this approach the young women were assisted to resolve their life challenges. Table 1 indicates the challenges that were identified and guidelines to address them.

**TABLE 1:** Identified challenges and guidelines to address challenges.

Identified challenges	Guidelines
**Intrapersonal challenges**	
Low self-concept leading to: academic failure sexual misbehaviour depression suicidal ideation.	Facilitation of mindfulness
**Interpersonal challenges**	
Inability to form and sustain relationships. Lives conflicted because of tumultuous relationship with mothers. Experience mothers not being there for them during painful times.	Facilitation of constructive interpersonal skills: Constructive communication. Assertiveness. Conflict management.

Mindfulness should be facilitated in these young women, who should become aware and discover themselves. This includes these young women needing to know themselves and understand their own thoughts, feelings and actions. Self-awareness (Johnson 2009:52–53; Uys & Middleton 2004:185–186) can lead the young women to behave pro-actively as well as responsibly in choosing constructive behaviour. The young women need to perceive their experiences in a non-judgemental way and not label their experiences in any way. They need to be compassionate towards themselves. Young women need to live in the present moment and accept that they are active participants and can choose to respond constructively to their perceived rejection from their mothers. Young women need to be open to new possibilities (Duke Health Organisation 2010) and see their own experiences as an opportunity to learn and grow.

To be able to form and sustain interpersonal relationships it is necessary for young women to practice their interpersonal skills. The young women also need to practice empathy – that is, to place themselves in the shoes of their mothers. They need to listen respectfully (Johnson 2009:234) and hear what their mothers say and then try and identify and understand their mother's point of view (Davidson & Wood 2004). It is also important to question feelings as well as facts. The young women also need to share their feelings (Foundation Coalition 2013) of rejection with their mothers. In addition, they need to manage their feelings (Foundation Coalition 2013) towards their mothers, and even leave the situation for a while to take control of their emotions.

## Conclusion

Our research study granted only a few young women an opportunity to vent their feelings and emotions regarding perceived rejection from their mothers. Very possibly there could be many more life stories to tell based on this phenomenon. Our hopes and wishes are not only that more research studies be done, but for other solutions to be determined and implemented. This, we believe, can better the lives of individuals and improve their mental, emotional, psychological and physical health.

## References

[CIT0001] Alaszewski A., 2006, *Using diaries for social research*, Sage Publications, London.

[CIT0002] Bornstein M.H., Davidson L., Keyes C.L.M. & Moore K.A. (Eds.), 2003, *Well-being: Positive development across the life course*, Lawrence Erlbaum Associates, London.

[CIT0003] Brewer M.B. & Hewstone M. (Eds.), 2004, *Self and social identity*, Blackwell Publishing, Malden.

[CIT0004] Burns N. & Grove S.K., 2005, *The practice of nursing research: Conduct, critique, and utilization*, 5th edn., Elsevier Saunders, St. Louis.

[CIT0005] Chambliss D.F. & Schutt R.S., 2010, *Making sense of the social world: Methods of investigation*, Pine Forge Press, Thousand Oaks, CA.

[CIT0006] Coleman J.W. & Kerbo H.R., 2006, *Social problems*, 9th edn, Pearson Education, Los Angeles.

[CIT0007] Cottle T.J., 2003, *Beyond self-esteem: Narratives of self-knowledge* & * devotion to others*, Peter Lang Publishing, New York.

[CIT0008] Creswell J.W., 2013, *Qualitative inquiry* & * research design: Choosing among five approaches*, 3rd edn., Sage Publications, London.

[CIT0009] Davidson J. & Wood C., 2004, ‘A conflict resolution model’, *Theory and Practice* 43(1), 6–13. 10.1353/tip.2004.0005

[CIT0010] Deeba F., 2001, *Home environment and parental acceptance-rejection and authorisation in child abuse*, Quaid-i-Azam University, Islamabad.

[CIT0011] Dhai A. & McQuoid-Mason D., 2011, *Bioethics, human rights, and health law: Principles and practice*, Juta & Company, Claremont.

[CIT0012] Duke Health Organisation, 2010, *How to bring more mindfulness in your life*, viewed 11 March 2013, from http://www.dukehealth.org/health_article/howtobringmoremindfulness

[CIT0013] Foundation Coalition, 2013, *Effective interpersonal/intra-team communication*, viewed 11 March 2013, from http://www.foundationcoalition.org.

[CIT0014] Gorman G.E. & Clayton P., 2005, *Qualitative research for the information professional: A practical handbook*, 2nd edn, Facet Publishing, London.

[CIT0015] Gray P.S., Williamson J.B., Karp D.A. & Dalphin J.R., 2007, *The research imagination: An introduction to qualitative and quantitative methods*, Cambridge University Press, New York 10.1017/CBO9780511819391

[CIT0016] Gull S.A., Tanq H. & Malik S., n.d., *Parental acceptance and rejection theory (part) assignment No. 1*, viewed 12 September 2009, from http.www.scribd.com/doc/16954509/Parental-Acceptance-and-Rejection-Theory.

[CIT0017] Hartley-Brewer E., 2001, *100 Tips for parents and teachers: Raising confident girls*, Fisher Books, Cambridge.

[CIT0018] Jay, n.d., *Helium testimonies: Dysfunctional mother and daughter relationships*, viewed 12 September 2009, from http://www.helium.com/items/869254-testimonies-dysfunctional-mother-and-daughter-relationships

[CIT0019] Johnson D.W., 2009, *Reaching out: Interpersonal effectiveness and actualisation*, Allyn & Bacon, Boston.

[CIT0020] Lessesne C.A. & Kennedy C., 2005, ‘Starting early: Promoting the mental health of women and girls throughout the life span’, *Journal of Women*’*s Health* 60(9), 754–763. 10.1089/jwh.2005.14.754 16313205

[CIT0021] Long T. & Johnson M., 2007, *Research ethics in the real world: Issues and solutions for health and social care*, Churchill Livingstone Elsevier, St Louis.

[CIT0022] Mark A., n.d., *Mother-daughter relationships: Reaping its positive benefits*, viewed 20 September 2009, from http://knol.google.com/k/abi-mark/mother-daughter-relationship-reaping/o1182174iwo5/90

[CIT0023] Marsh H.W., Craven R.G. & McInerney D.M. (Eds.), 2003, *International advances in self research*, Information Age Publishing, Connecticut.

[CIT0024] McAdams D.P., 2009, *The person: An introduction to the science of personality psychology*, 5th edn., John Wiley & Sons, Hoboken.

[CIT0025] Miller A.G. (ed.), 2004, *The social psychology of good and evil*, Guilford Press, New York.

[CIT0026] Mind for better mental health, 2009, *Women and mental health*, viewed 16 July 2009, from http//www.mind.org.uk/Information/Factsheets/Women+and+mental+ health.htm

[CIT0027] Polit D.F. & Beck C.T., 2010, *Essentials of nursing research: Appraising evidence for nursing practice*, 7th edn., Lippincott Williams & Wilkins, Philadelphia.

[CIT0028] Rholes W.S. & Simpson J.A. (eds.), 2004, *Adult attachment: Theory, research, and clinical implications*, The Guilford Press, New York.

[CIT0029] Rohner R.P. & Khaleque A., n.d., *Parental acceptance-rejection and life-span developments: A universal perspective*, viewed 12 September 2009, from http://www.scribd.com/doc/16954509/Parental-Acceptance-and-Rejection-Theory

[CIT0030] Sadock B.J. & Sadock V.A., 2007, *Kaplan* & * Sadock*’*s synopsis of psychiatry: Behavioural sciences/clinical psychiatry*, 10th edn, Lippincott Williams & Wilkins, Philadelphia.

[CIT0031] Sedikides C. & Spencer S.J. (Eds.), 2007, *The self*, Psychology Press, New York.

[CIT0032] Shaffer D.A., 2005, *Social and personality development*, 5th edn., Thomson Wadsworth, Bellmont.

[CIT0033] Shenton A.K., 2004, *Strategies for ensuring trustworthiness in qualitative research projects*, viewed 03 March 2011, from http://iospress.metapress.com/content/3ccHm2g59cklapx/

[CIT0034] Stuart B., 2009, *The mother-daughter relationship*, viewed 12 September 2009, from http://www.selfgrowth.com/print/667598

[CIT0035] *The Oxford Advanced Learner*’*s Dictionary*, 2011, ‘Experience’, viewed 3 March 2012, from http://www.theoxfordadvancedlearnersdictionary.com/p/experience.

[CIT0036] Ty E. & Verduyn C. (Eds.), 2008, *Asian Canadian writing beyond autoethnography*, Wilfrid Laurier University Press, Waterloo, Canada.

[CIT0037] Uys L. & Middleton L. (Eds.), 2004, *Mental health nursing: A South African perspective*, 4th edn, Juta & Co, Cape Town.

[CIT0038] Williams K.D., Forgas J.D. & Von Hippel W. (Eds.), 2005, The social outcast: Ostracism, social exclusion, rejection, and bullying, Psychology Press, New York.

[CIT0039] World Health Organization, 2004, *Promoting mental health: Concepts, emerging evidence, practice*, World Health Organization Publications, Geneva.

